# Virulence and antimicrobial resistance gene profiles of *Staphylococcus aureus* associated with clinical mastitis in cattle

**DOI:** 10.1371/journal.pone.0264762

**Published:** 2022-05-03

**Authors:** Neelam, V. K. Jain, Mahavir Singh, Vinay G. Joshi, Rajesh Chhabra, Kuldeep Singh, Y. S. Rana

**Affiliations:** 1 Department of Veterinary Medicine, COVS, Lala Lajpat Rai University of Veterinary and Animal Sciences, Hisar, Haryana, India; 2 College Central Laboratory, COVS, Lala Lajpat Rai University of Veterinary and Animal Sciences, Hisar, Haryana, India; 3 Department of Animal Biotechnology, COVS, Lala Lajpat Rai University of Veterinary and Animal Sciences, Hisar, Haryana, India; The Rockefeller University, UNITED STATES

## Abstract

*Staphylococcus aureus* (*S*. *aureus*) is the most prevalent microorganism associated with mastitis in cattle, which harbours several virulence factors and antibiotic resistance genes. The present study aimed to characterize *S*. *aureus* isolated from mastitic milk of the cattle for antibiotic resistance (*blaZ* and *mecA)*, haemolysins (*hla* and *hlb*) and enterotoxins (*sea*, *seb*, *sec*, and *sed*) genes. A total of 69 staphylococci were isolated and phenotypically characterized for haemolytic properties on 5% sheep blood agar medium. Out of 69 isolates, 55 (79.71%) were identified as *S*. *aureus* by polymerase chain reaction assay. Among *S*. *aureus*, the majority of the isolates harboured the gene *blaZ* (92.73%), followed by *coa* (89.09%), *hlb* (60%) and *hla* (49.09%). Gene *mecA* responsible for methicillin resistance was detected in 23.64% of *S*. *aureus* isolates. Enterotoxin genes *seb* (9.09%), *sec* (1.82%) and *sed* (7.27%) responsible for food poisoning were detected at a comparatively lower rate and none of the *S*. *aureus* strain was found positive for *sea*. Additionally, antimicrobial susceptibility study of *S*. *aureus* against 18 antimicrobial discs showed maximum resistance to oxytetracycline, penicillin, and fluoroquinolone groups, contrarily, we observed maximum sensitivity to methicillin and cefuroxime antimicrobials. The high occurrence rate of *S*. *aureus* harbouring genes for virulence factors and antimicrobial resistance needs appropriate strategies to control the pathogen spread to the human population.

## Introduction

*Staphylococcus aureus* (*S*. *aureus*) is one of the major food-borne pathogens associated with various diseases in human beings and animals [1]. Staphylococci are the most common aetiological agent of mastitis in cattle causing deterioration of both milk quality and quantity [2]. The pathogenicity of this organism is mainly attributed to invasive function, biofilm formation, toxin-mediated virulence factors and antimicrobial resistance [3–5]. *S*. *aureus* produces various types of virulence factors like haemolysins, leukocidins, enterotoxins, superantigens which lead to the development of intramammary infection and help the pathogen to escape from the host immune system [6]. Four different types of haemolysins, namely *α*, *β*, *γ* and δ toxins are produced by the organism; of which; *α* and *β* play a major role in the pathogenicity of *S*. *aureus* [3]. Enterotoxins secreted by staphylococci are short, proteolytic enzymes that remain active in the gastrointestinal system after ingestion and subsequently act on specific emetic receptors. Till now, 23 serologically distinct staphylococcal enterotoxins have been identified [7]. Out of the identified enterotoxins; sea, seb, sec, sed and see represent the “classical types” and are well characterized [8]. Staphylococcal enterotoxins are resistant to destruction by heat treatment and may retain their biological activity [9]. The consumption of milk and milk products having staphylococcal enterotoxins may cause food poisoning [1].

Another prime public health concern is the detection of antibiotic-resistant strains in cases of bovine mastitis and the possibility of transmission to human beings via the consumption of unpasteurized dairy products. Irrational use of antibiotics in mastitis treatment may lead to the proliferation of resistant strains and drug residues in milk also pose serious community health concerns [10]. The biofilm formation potential of staphylococci in udder parenchyma further aggravates the antibiotic resistance problem. Some exotoxins like alpha haemolysin and Leukotoxin AB secreted by staphylococci also play a pre-eminent role in the development of biofilm [11]. Antibiotic sensitivity test is an essential assay to develop a cautious and sensible approach towards the use of antimicrobials for the treatment of mastitis in animals [12]. The objective of the present study was the isolation and identification of *S*. *aureus* in milk samples from the clinical mastitis cases and to understand the involvement of virulence genes associated with food safety. In addition to the screening of bacterial isolates for the virulence genes using PCR, we performed antibiogram studies with molecular profiling. The study underlined the importance of molecular and phenotypic antibiotic resistance profiling for routinely used antibiotics for improvising management and therapeutic practices.

## Materials and methods

### Ethics statement

The milk samples used in the study were received in the laboratory from the animal owners for bacterial isolation and antibiotic sensitivity testing. The history of the animal with the symptoms of mastitis was recorded at the time of sample submission. The milking/milk sample collection procedure does not involve invasive procedures therefore ethical permissions are not indicated. Verbal consent was obtained from the animal’s owners for the samples under study.

### Bacterial isolation and phenotypic characterization

A total of 565 quarter milk samples of 142 dairy cattle suffering from clinical mastitis were received at the College Central Laboratory and processed for bacterial isolation. The samples were thoroughly mixed and 10 μl of milk was inoculated on 5% defibrinated sheep blood agar plates [13]. After incubation of the plates at 37° C for 16–18 h, the bacterial colonies were identified by gross morphology, Gram’s staining and catalase test. Haemolytic activity of staphylococci was recorded on 5% sheep blood agar plates. Alpha haemolysis was observed after overnight incubation of plate at 37°C. To identify beta haemolysis, plates incubated at 37°C showing a wide hazy zone of haemolysis, were kept at 4°C in the refrigerator and observed for turning off the wide hazy zone into a clear zone of haemolysis [13].

### Confirmation of *S*. *aureus* by PCR assay

Genomic DNA of phenotypically identified colonies of *Staphylococci* spp. was extracted by commercially available DNeasy® Blood & Tissue Kit (Qiagen, Germany), according to the manufacturer’s instructions and was stored at -20°C till further analysis.The *16S rRNA* and *23S rRNA* genes were targeted to confirm *Staphylococcus* spp. and *S*. *aureus*, respectively. The commercially available master mix (2x HotStarTaq Plus Master Mix; Qiagen, Germany) was used to set up the PCR reaction. The reaction mixture used in this study was as follows: 2.5 μl of DNA was added to 22.5 μl of PCR mixture of 2x HotStarTaq Plus Master Mix (Qiagen, Germany) and 0.2 μM of each forward and reverse primer (Table 1). Target gene amplification was done by PCR using Thermal Cycler (Biorad T100^TM^). Thermal cycling conditions were optimized with initial denaturation at 95°C for 5 min, 30 cycles (denaturation, 95°C, 1 min; annealing, 55°C, 1 min; extension, 72°C, 1 min), final extension at 72°C for 10 min and held at 4°C. Non-template control (NTC) and positive control (DNA from *Staphylococcus aureus* ATCC strain 700699, HiMedia, Mumbai) were used to check the reliability of the PCR reactions.

**Table 1:** Oligonucleotide primers used in the study.

Factor	Gene	5’——-sequence of primer sets———3’	Product size (bp)	Reference
Genus-specific	*16S rRNA*	**F**-GCAAGCGTTATCCGGATTT **R**-CTTAATGATGGCAACTAAGC	597	[14]
Species-specific	*23S rRNA*	**F-**GGACGACATTAGACGAATCA **R**-CGGGCACCTATTTTCTATCT	1319	[15]
Coagulase	*coa*	**F-**ACCACAAGGTACTGAATCAACG **R**-TGCTTTCGATTGTTCGATGC	750	[16]
Haemolysins	*hla*	**F-** GGTTTAGCCTGGCCTTC **R-** CATCACGAACTCGTTCG	550	[17]
	*hlb*	**F-** GCCAAAGCCGAATCTAAG **R-** GCGATATACATCCCATGGC	840	[17]
Enterotoxins	*sea*	**F-** GCAGGGAACAGCTTTAGGC **R-** GTTCTGTAGAAGTATGAAACACG	521	[18]
	*seb*	**F-** ACATGTAATTTTGATATTCGCACTG **R-** TGCAGGCATCATGTCATACCA	667	[18]
	*sec*	**F-** CTTGTATGTATGGAGGAATAACAA **R-** TGCAGGCATCATATCATACCA	284	[18]
	*sed*	**F-** GTGGTGAAATAGATAGGACTGC **R-** ATATGAAGGTGCTCTGTGG	385	[18]
Antibiotic resistance genes	*mecA*	**F-** AAAATCGATGGTAAAGGTTGGC **R-** AGTTCTGCAGTACCGGATTTGC	532	[19]
	*blaZ*	**F-** ACTTCAACACCTGCTGCTTTC **R-** TGACCACTTTTATCAGCAACC	173	[20]

### PCR assay for the detection of virulence factors and antibiotic resistance genes of *S*. *aureus*

The isolates which were confirmed as *S*. *aureus* by species-specific PCR were further screened by PCR assay for the presence of coagulase (*coa*), alpha haemolysin (*hla*), beta haemolysin (*hlb*), staphylococcal enterotoxin A (*sea*), staphylococcal enterotoxin B (*seb*), staphylococcal enterotoxin C (*sec*), staphylococcal enterotoxin D (*sed*) virulence genes along with beta-lactam resistance (*blaZ*) and methicillin (*mecA*) gene. Sequences of the primers are listed in Table 1. The reaction mixture and thermal cycling conditions were used as mentioned in earlier section except for *blaZ* amplification where the annealing temperature was 50°C. All reactions included positive control and NTC. All the amplified products were resolved in 1.5% agarose gel, in 1x TAE buffer with the definite length of molecular marker for approximately 2 h under 70 volt. The amplified PCR products were visualized under a gel documentation system (Azure^TM^ 150C gel documentation, USA).

#### Sequencing of amplified products

Purification of PCR amplified products was done by using QIAquick gel extraction kit (Qiagen, Germany) as per the instructions of the manufacturer. The purified PCR products were further subjected to sequencing using Automated DNA Sequencer Applied Biosystem 3130XL Genetic analyser (USA) and the sequences were submitted to NCBI.

### Antimicrobial susceptibility testing of *S*. *aureus*

Antimicrobial susceptibility testing of *S*. *aureus* isolates was done by the disc diffusion method on Mueller-Hinton agar, using commercially available antibiotic discs as per the method of Markey et al. [21]. A total of 18 antimicrobial discs (HiMedia, Mumbai) with their specified concentration (in parenthesis); penicillin (10 units), amoxicillin (10 μg), ampicillin (10 μg), cloxacillin (30 μg), oxacillin (5 μg), methicillin (5 μg), cefuroxime (30 μg), cefoperazone (75 μg), ceftriaxone (30 μg), cefotaxime (30 μg), amikacin (30 μg), gentamicin (10 μg), chloramphenicol (30 μg), enrofloxacin (10 μg), levofloxacin (5 μg), moxifloxacin (5 μg), oxytetracycline (30 μg) and lincomycin (10 μg) were used in the study. Bacterial isolates were classified as susceptible, intermediate, or resistant according to the manufacturer’s instructions.

## Results

### Bacterial isolation and identification

Sixty-nine bacterial isolates were identified as *Staphylococcus* spp. based on gross morphology on blood agar, positive catalase test and Gram-positive cocci in bunches on microscopic examination. These colonies of staphylococci were further confirmed by genus specific PCR. Out of 69 isolates, 55 were confirmed as *S*. *aureus* by species-specific PCR; of which nine (16.36%) and 14 (25.45%) isolates exhibited α haemolytic and β haemolytic activity on blood agar plates, respectively.

### Detection of virulence factors and antibiotic resistance genes in *S*. *aureus*

The frequency of genes for coagulase, haemolysins, enterotoxins and genes responsible for antibiotic resistance in *S*. *aureus* is depicted in Table 2. Out of 55 *S*. *aureus* isolates, 89.09% (49/55) were found to be positive for the coagulase gene. Alpha-haemolysin and beta-haemolysin genes were present in 49.09% (27/55) and 60% (33/55) of the isolates, respectively. None of the isolate was found positive for the *sea* gene. However *seb*, *sec* and *sed* genes were detected in 9.09% (5/55), 1.82% (1/55) and 7.27% (4/55) of *S*. *aureus* isolates, respectively. Gene responsible for antibiotic resistance against β-lactam antibiotics, *blaZ* was detected in 92.73% isolates (51/55).The *mecA* gene responsible for methicillin resistance was detected in 23.64% (13/55) of *S*. *aureus* isolates.

**Table 2:** Detection of virulence factors and antibiotic resistance genes in *S*. *aureus* (n = 55) isolates.

Factors	Genes	n	Percentage
Coagulase	*coa*	49	89.09
Haemolysins	*hla*	27	49.09
	*hlb*	33	60.00
Enterotoxins	*sea*	0	0.00
	*seb*	5	9.09
	*sec*	1	1.82
	*sed*	4	7.27
Antibiotic resistance genes	*blaZ*	51	92.73
	*mecA*	13	23.64

n = Number of isolates found positive for a particular gene

#### Accession number(s)

Sequencing results of amplified products of the genes submitted to NCBI were assigned the following accession numbers: *blaZ;* MT588201, *mecA;* MT780102, *hla;* MT780101, *hlb;* MT780104, *seb;* MT780105 and *sec;* MT780107.

### Antimicrobial susceptibility testing of *S*. *aureus*

Antimicrobial susceptibility testing of *S*. *aureus* (Table 3) revealed the varying extent of resistance against the drugs of the same and different classes of antibiotics. The highest resistance was observed against oxytetracycline (98.18%) and most of the antimicrobials of the Penicillin group, [ranging from cloxacillin (96.36%) to penicillin (83.64%)]. However, lowest resistance (12.73%) was exhibited against methicillin. On the other hand, among cephalosporins, the highest resistance was exhibited against cefoperazone (47.27%), while the least resistance was observed against cefuroxime (21.82%). Among aminoglycosides, 16.36% of *S*. *aureus* isolates were sensitive to amikacin, while none of the isolate was sensitive to gentamicin. Most of the *S*. *aureus* isolates were found resistant against fluoroquinolones [enrofloxacin (90.91%), moxifloxacin (89.09%) and levofloxacin (70.91%)]. Most of the *S*. *aureus* were intermediate to chloramphenicol (45.45%), and resistant to lincomycin (49.09%).

**Table 3:** Antimicrobial susceptibility of *S*. *aureus* (n = 55) isolated from clinical cases of cattle.

Antibiotic class	Antimicrobial drug	Antimicrobial susceptibility					
		Resistant		Intermediate		Sensitive	
		n	%	n	%	n	%
Penicillins	Penicillin	46	83.64	0	0.00	9	16.36
	Amoxicillin	49	89.09	0	0.00	6	10.91
	Ampicillin	49	89.09	0	0.00	6	10.91
	Cloxacillin	53	96.36	0	0.00	2	3.64
	Oxacillin	52	94.55	0	0.00	3	5.45
	Methicillin	7	12.73	11	20.00	37	67.27
Cephalosporins	Cefuroxime	12	21.82	10	18.18	33	60.00
	Cefoperazone	26	47.27	20	36.36	9	16.36
	Ceftriaxone	17	30.91	22	40.00	16	29.09
	Cefotaxime	13	23.64	27	49.09	15	27.27
Aminoglycosides	Amikacin	32	58.18	14	25.45	9	16.36
	Gentamicin	19	34.55	36	65.45	0	0.00
Amphenicols	Chloramphenicol	21	38.18	25	45.45	9	16.36
Fluoroquinolones	Enrofloxacin	50	90.91	0	0.00	5	9.09
	Levofloxacin	39	70.91	7	12.73	9	16.36
	Moxifloxacin	49	89.09	4	7.27	2	3.64
Tetracyclines	Oxytetracycline	54	98.18	0	0.00	1	1.82
Lincosamides	Lincomycin	27	49.09	0	0.00	28	50.91

n = number of isolates

## Discussion

Monetary losses in dairy farming occur mainly due to poor management of udder health. Managemental flaws are prime attributes of bacterial contamination of mammary secretions [22]. *S*. *aureus* is the most prevalent bacterial species responsible for mastitis in cattle. Virulence factors such as enterotoxins and haemolysins produced by staphylococci in the milk could generate potential public health implications.

In the present study, we examined the presence of *S*. *aureus* associated with cattle mastitis with a comprehensive study of virulence factors and antibiotic resistance patterns in the isolates. *S*. *aureus* is one of the most commonly reported bacteria in cases of cattle mastitis. The high prevalence of *S*. *aureus* in this study may be due to customary hand milking practice, absence of regular post milking teat dip and lack of knowledge about dry cow therapy among the dairy owners. Transmission of staphylococci from infected to healthy udder quarters mainly occurs between animals during the milking process through the milker’s hands [2, 23].

Coagulase production is a considerable phenotypic feature, used to identify *S*. *aureus*. However, the phenotypic method is insufficient for the differentiation of coagulase-positive and negative isolates [24]. Therefore, we examined the isolates for the *coa* gene by PCR. In our study, we observed a mixed pattern with large numbers of coagulase-positive *S*. *aureus* (89.09%) along with a minor number of coagulase-negative isolates (10.91%). However, the coagulase gene was present in all the isolates of staphylococci in the research carried out by Elsayed et al. [25] and Xu et al. [26]. Many researchers [27–30] reported the association of coagulase-negative staphylococci with bovine mastitis and considered them emerging mastitis pathogens.

The *S*. *aureus* isolates detected from mastitic milk samples were screened for the presence of virulence and antibiotic resistance genes by PCR. The *hla* expression is associated with the production of toxin alpha-haemolysin, which is known to cause the gangrenous type of mastitis involving restriction of blood circulation to mammary tissues and resultant damage to smooth muscles [31]. Gene *hlb* is associated with the production of the toxin beta-haemolysin, which is neutral sphingomyelinase [32] that can degrade sphingomyelin in cell membranes of erythrocytes, leukocytes, neurons and other tissue cells [33, 34]. β-haemolysin leads to biofilm formation [35] and is responsible for more adherence of *S*. *aureus* to bovine mammary gland epithelium and increased tolerance to antimicrobials [6]. The occurrence of *hla* (49.09%) and *hlb* (60%) genes in *S*. *aureus* isolates in the present study are in close agreement with the findings of Elsayed et al. [25], Rodrigues et al. [36] and Dan et al. [37], while Yang et al. [38], Xu et al. [26] and Wang et al. [39] reported a higher percentage of *hla* and *hlb* in *S*. *aureus* isolates. In the present study, phenotypically, only 25.45% and 16.36% *S*. *aureus* isolates exhibited beta and alpha haemolysis on sheep blood agar plates, respectively. The expression of virulence genes is regulated by various regulatory systems along with growth conditions and growth phases.

*S*. *aureus* strains are also considered significant food borne pathogens mainly due to the production of enterotoxins, which further contribute to the pathogenesis of various human diseases like toxic shock syndrome, pneumonia, sepsis, food poisoning outbreaks etc. The role of enterotoxins in the pathogenesis of bovine mastitis remains ambiguous, but their presence in milk poses a serious public health concern. Enterotoxins are stable at high temperature and retain their biological activity in milk even after pasteurization [40]. Infected udder of animals with enterotoxigenic staphylococci become the source of enterotoxins in milk which may lead to diarrhoea and other complications in human beings [41]. More than 90% of *S*. *aureus* associated food poisoning outbreaks were associated with the classical staphylococcal enterotoxins (denoted as SEA to SEE) [42]. Here, we screened all the *S*. *aureus* isolates for the presence of staphylococcal enterotoxin genes *sea*, *seb*, *sec* and *sed*. None of the *S*. *aureus* was found positive for the presence of the *sea* gene. In agreement with our study, Gomez et al. [43], Kumar et al. [44], Yang et al. [38], Rodrigues et al. [36] and Fursova et al. [45] could not detect *sea* gene in their study. Contrary to this, Xu et al. [26], Grispoldi et al. [46] and Monistero et al. [47] reported *sea* gene in 7.10%, 35.29% and 65.60% of the isolates, respectively.

Staphylococcus enterotoxin B is one of the most potent bacterial superantigens and contributes to the fatal exacerbation of community-associated methicillin-resistant *S*. *aureus* infection [48]. We detected five isolates of *S*. *aureus* harbouring the *seb* gene by PCR. The occurrence of *seb* (9.09%) is higher in the present study as compared to the findings of Kumar et al. [44], Rodrigues et al. [36] and Grispoldi et al. [46], wherein they detected the gene in 0.90%, 4.10% and 5.88% of *S*. *aureus* isolates, respectively. Contrary to this, Gomez et al. [43], Xu et al. [26] and Fursova et al. [45] could not detect *seb* in any of the *S*. *aureus* isolates in their respective studies.

Another virulence factor secreted by staphylococci is enterotoxin C which contributes to inflammatory reactions leading to the production of inflammatory cytokines and tissue damage in the mammary gland [49]. The percentage of *S*. *aureus* isolates having *sec* (1.82%) was lower in our study than Kumar et al. [44] and Grispoldi et al. [46] where they reported 8.40% and 5.88% of the *S*. *aureus* isolates, respectively, as *sec* positive. Contrary to this, Gomez et al. [43], Xu et al. [26], Rodrigues et al. [36] and Fursova et al. [45] could not detect *sec* in any of the *S*. *aureus* isolates in their studies. The role of enterotoxin D in mastitis has been studied by Tollersrud et al. [50] and they observed that this toxin is secreted in mammary secretions and stimulates specific antibody responses in cows in the course of experimental intramammary infections. Our study revealed higher number of *S*. *aureus* isolates (7.27%) harbouring *sed* than Kumar et al. [44], but lesser as compared to the findings of Grispoldi et al. [46] where they detected 0.90% and 29.41% of isolates as *sed* positive respectively. Contrary to this, Xu et al. [26] and Rodrigues et al. [36] could not detect *sed* in any of the *S*. *aureus* isolates in their respective studies.

In the present scenario, antibiotic resistance is a major challenge to human and livestock health and have been reported worldwide with all microbes showing different resistance levels to a vast majority of antimicrobials [51]. The same phenomenon has been observed in the present study as well. The finding of beta-lactam resistance gene *blaZ* in 92.73% *S*. *aureus* isolates in the study; is in close agreement with Yang et al. [52], who reported the presence of *blaZ* in 95.45% of isolates from the bovine mastitis cases. Xu et al. [26] reported a slightly lower presence of *blaZ* (82.10%) compared to the present study. However, contrary to this, Piotr et al. [53] and Monistero et al. [47] reported only 20.32% and 46.20% *blaZ*. The higher occurrence of *blaZ* may be due to the choice of penicillin therapy for the treatment purpose over a long period, which may be responsible for selection pressure for *blaZ* harbouring strains of staphylococci.

Higher mortality rates are more associated with infections due to methicillin-resistant strains of *S*. *aureus* (MRSA) than infections caused by methicillin-susceptible strains. The worldwide presence of the MRSA in livestock is mainly attributed to the indiscriminate use of antimicrobial agents in animal husbandry and other agricultural activities [54]. Many researchers have identified MRSA from the milk of dairy animals suffering from mastitis; thus pointing towards a significant public health challenge. Methicillin resistance in *S*. *aureus* developed due to a mobile genetic element acquisition called the staphylococcal cassette chromosome *mec*. This cassette carries the *mecA* gene controlling the production of low-affinity penicillin-binding protein 2a and confers the pathogen resistance against the β-lactam antibiotics [55]. Our study revealed *mecA* in 23.64% of *S*. *aureus* isolates associated with cattle mastitis. Xu et al. [26] and Patel et al. [54] reported *mecA* in 35.70% and 73.08% of *S*. *aureus* isolates respectively. A lesser percentage of *mecA* was reported by Piotr et al. [53], Feng et al. [52], Rodrigues et al. [36], Dan et al. [37] and Monistero et al. [47] as compared to the findings of the present study. The differences in the emergence of different virulence factors in various studies may be due to variations in the managemental practices and agro-climatic conditions [56]. Phenotypically, out of 55 isolates from the present study, seven isolates were found resistant and 11 were intermediate towards methicillin by *in-vitro* antimicrobial susceptibility testing. Kulangara et al. [57] also reported phenotypic resistance to oxacillin in seven isolates of staphylococci with the absence of the *mecA* or *blaZ* gene.

All the *S*. *aureus* isolates in the study were subjected to *in-vitro* antimicrobial sensitivity testing. Phenotypically, *S*. *aureus* isolates showed high resistance to oxytetracycline, members of penicillin and fluoroquinolones group. The remarkable increase in antimicrobial resistance may be due to extensive use of the same class of antibiotics for treatment purposes and imprudent use of antimicrobials without prior antibiogram profiling of the causative agent [58].

The characterization of *S*. *aureus* is crucial for risk assessment due to its virulence properties involved in the mastitis disease process. The findings of our study regarding antimicrobial resistance genes and enterotoxin genes are significant because of the public health interests involved. Adoption of hygienic practices is necessary to control the pathogen spread from animal to animal. These practices also essential for the healthy and safe production of food for humans. As *S*. *aureus* from livestock may emerge as a threat to public health, the information generated here could be helpful for concerned veterinarians in improving dairy cattle health along with designing strategies for better and safe milk production.
